# Nanopore Electroporation: A New Delivery Method Within the Field of Epigenetic Editing

**DOI:** 10.1002/smll.202513858

**Published:** 2026-03-26

**Authors:** Frida Ekstrand, Sabrina Ruhrmann, Karl Bacos, Sabine Bartel, Pytrick Jellema, Marianne G. Rots, Charlotte Ling, Christelle N. Prinz

**Affiliations:** ^1^ Division of Solid State Physics and NanoLund Lund University Lund Sweden; ^2^ Department of Clinical Science in Malmö Epigenetics and Diabetes Unit Lund University, Scania University Hospital Malmö Sweden; ^3^ Department of Pathology and Medical Biology University of Groningen, University Medical Center Groningen Groningen the Netherlands; ^4^ SciLifeLab Lund University Lund Sweden

**Keywords:** diabetes, electroporation, epigenetic editing, nanopores, transfection

## Abstract

Epigenetic modifications influence gene expression and contribute to type 2 diabetes (T2D), but establishing causality requires targeted modulation of specific genes. CRISPR‐dCas9‐based tools offer this potential, yet β‐cells are notoriously difficult to transfect, and efficient, non‐viral delivery methods are lacking. Here, we developed nanopore‐mediated electroporation to deliver a CRISPR interference (CRISPRi) system to clonal INS1 β‐cells, achieving targeted downregulation of insulin expression. Cells were seeded atop a nanopore substrate with CRISPRi plasmids in solution below. Mild electric pulses generated transient nanoscale pores in the membrane, enabling electrophoretic delivery of plasmids into the cytosol while preserving high cell viability. The CRISPRi system comprised the transcriptional repressor Krueppel‐associated Box Domain (KRAB) fused to an inactive Cas9 (dCas9), guided to the transcription start site of the insulin‐1 gene (*Ins1*) by a single guide RNA (sgRNA). After transfection, *Ins1* expression was significantly reduced, demonstrating effective modulation of gene expression in this difficult‐to‐transfect cell type. This nanopore electroporation approach provides a robust, safe, and efficient platform for delivering CRISPR‐dCas9‐based epigenetic editors in pancreatic β‐cells. By enabling precise gene regulation, it opens avenues for mechanistic studies of epigenetic contributions to T2D and potentially other challenging cell systems.

## Graphical abstract

Nanopore‐mediated electroporation was used to deliver a CRISPRi system to clonal β‐cells. This enabled targeted downregulation of insulin expression while maintaining high cell viability.

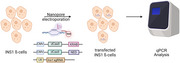

## 1 Introduction

The number of people developing age‐related complex diseases, including T2D, is rapidly increasing worldwide [1]. T2D is characterized by chronically elevated blood glucose levels caused by impaired insulin secretion from the pancreatic β‐cells in combination with insulin resistance in target tissues, such as the liver, adipose tissue and skeletal muscle. In non‐diabetic people, pancreatic β‐cells secrete insulin in response to a meal. However, molecular defects have been identified in β‐cells from people with T2D, contributing to a deficient β‐cell response to elevated glucose, and subsequently impaired insulin secretion [2, 3, 4]. A combination of genetic, epigenetic and environmental factors contributes to the development of T2D [5, 6]. Epigenetic mechanisms include DNA methylation, histone modifications and non‐coding RNAs [7]. These shape the 3D structure of the genetic material and regulate cell‐specific gene expression. As epigenetic alterations are important mechanisms in complex diseases, they may offer potential novel therapeutic targets [8]. For example, we have discovered epigenetic modifications in tissues from people with, or at risk for, T2D compared with non‐diabetic controls [9, 10, 11, 12, 13, 14, 15, 16, 17], including changes in pancreatic islets that contribute to the impaired insulin secretion that is a hallmark of T2D [13, 14, 15, 18, 19, 20, 21]. However, it has been a challenge to establish whether disease‐associated epigenetic modifications cause the disease, or if the disease causes the observed modifications in epigenetic marks [5, 13].

To identify epigenetic changes with causal effects on phenotypes contributing to disease development, one can use epigenetic editing to deposit or remove epigenetic modifications onto a specific genomic region and analyze whether this alters gene expression and affects cellular processes. This can be done stably both in vitro and in vivo [22, 23, 24].  The CRISPR‐dCas9 system has emerged as a promising method for epigenetic editing [25, 26]. It relies on the delivery of a sgRNA, used for targeting a genomic region of interest, together with a catalytically deactivated Cas9 protein (dCas9) fused to e.g., artificial transcription factors or catalytic domains of epigenetic enzymes, such as DNA methyltransferases (DNMTs) [27] and Ten Eleven Translocation enzymes (TETs) [28]. CRISPR interference (CRISPRi) uses dCas9 fused to a repressor, e.g., the non‐catalytic transcriptional repressor Krueppel‐associated Box Domain (KRAB), thus downregulating the target gene [29]. Epigenetic editing opens up for potentially correcting unfavourable epigenetic traits in a disease state.

To regulate the expression of selected candidate genes using a CRISPRi system in cell lines, these have generally been transfected with DNA plasmids coding for the components described above [30, 31]. However, currently used transfection methods have drawbacks that limit efficiency in many cell types, including clonal β‐cells. For example, chemical methods, such as lipofectamine, are prone to endosomal entrapment [32, 33] which hinders the cargo from reaching its destination. Viral transduction faces the risk of mutagenesis and can cause inflammatory responses [34, 35]. Bulk electroporation uses strong electric fields to destabilize the cell membrane of cells in suspension so that the cargo can diffuse into the cells. The strong electric fields can cause irreversible damage to the cell membrane, lowering the cell viability [36, 37]. A non‐exhaustive list of transfection methods, along with their advantages and disadvantages, can be found in Table S1.

**FIGURE 1:**
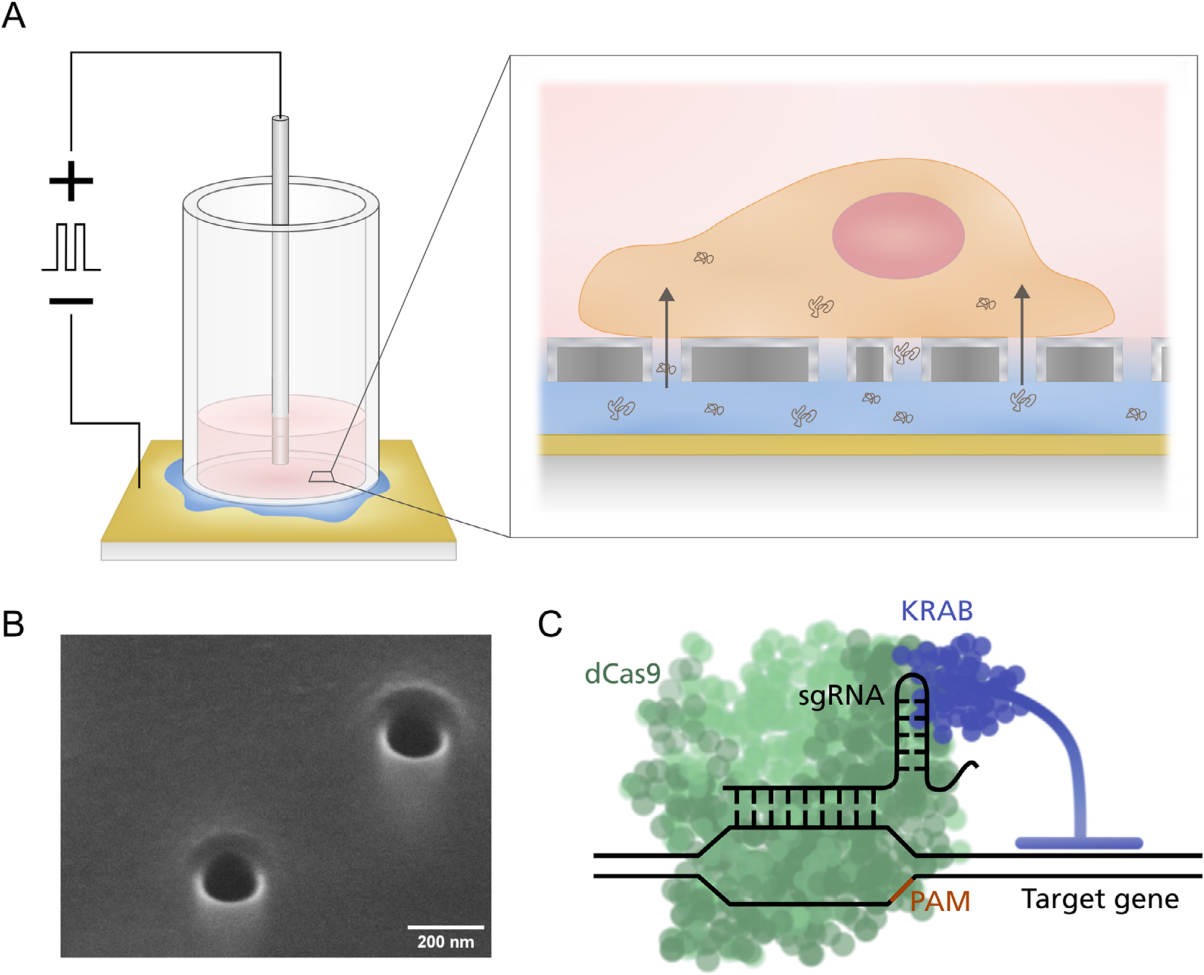
CRISPR‐dCas9‐mediated editing of the *Ins1* gene (encoding insulin) in rat clonal β‐cells using nanoelectroporation. (A) Schematics of the nanopore electroporation device. Cells are seeded on the nanopore substrate at the bottom of a plastic cylinder. The device is placed on top of a drop of plasmid solution deposited on a gold‐coated microscope slide, which serves as the bottom electrode. A Pt‐electrode is placed in contact with the cell medium in the plastic cylinder, and trains of square electrical pulses are applied between the two electrodes. The electrical pulses locally open the cell membrane on top of the nanopores and drive the plasmids to the cytosol (inset). The sketch is not to scale. (B) Scanning electron microscope (SEM) image of alumina coated nanopores with an original nominal pore diameter of 200 nm. In‐lens detector, 30° stage tilt. (C) Schematic representation of the dCas9 with the transcriptional repressor KRAB and the sgRNA bound to the region of interest on the DNA. The dCas9 searches for the protospacer adjacent motif (PAM) sequence and tests whether the sgRNA matches the downstream DNA sequence. If there is a match, the whole complex is fixed in that location, and the KRAB hinders the expression of the gene of interest.

Nanoelectroporation is an alternative to traditional transfection methods for use in vitro or ex vivo and has been studied for some cell types and applications [38, 39, 40, 41]. Cells are seeded on top of a porous plastic substrate (either flat or with protruding nanostraws), with pore diameters in the range of hundreds of nanometers, with the cargo solution below the substrate (Figure 1A). Upon application of a train of electric pulses across the cells and substrate, the pores concentrate the field lines, resulting in membrane disruption that is minor relative to the overall cell size. If the polarity of the electric field is chosen correctly, in accordance with the cargo molecule charge, electrophoresis helps transport the cargo to the cytosol and increases the transfection efficiency significantly [42, 43]. This effect is especially important for larger molecules, such as plasmids, where diffusion is slow. Compared to bulk electroporation, when using nanoelectroporation, the electric field is focused on a much smaller area, therefore the cell viability is higher, and the stress response is reduced [44, 45, 46]. The method is amenable to multiplexing and has been used to deliver nanoparticles, oligonucleotides, proteins, plasmids and ribonucleoproteins in cells [38, 39, 47, 48, 49, 50, 51]. However, to our knowledge, nanoelectroporation has not been developed to successfully transfect cells with the large CRISPR‐dCas9 system to achieve gene silencing or epigenetic editing. Based on this background, the goal of this study was to establish and validate nanoelectroporation as a non‐viral delivery method for large CRISPR‐dCas9‐based editing systems in rat clonal β‐cells, and to determine whether this approach enables efficient CRISPRi‐mediated downregulation of *Ins1*, encoding for insulin.

Here, we report a novel study, demonstrating that nanoelectroporation enables CRISPR‐dCas9‐mediated downregulation of *Ins1* in rat INS1 832/13 β‐cells (hereafter called INS1 β‐cells), thus paving the way for epigenetic editing using nanoelectroporation as a delivery method (Figure 1B). The CRISPRi system consists of one plasmid encoding the sgRNA and one plasmid encoding dCas9‐KRAB. Importantly, these two plasmids are of similar size (3500 and 8000 kb, respectively) as other plasmids available for epigenetic editing [52]. After transcription (and translation of dCas9‐KRAB), the sgRNA and dCas9‐KRAB form a complex, where the guide ensures that the complex is targeted to the correct genomic location to downregulate the gene of interest (Figure 1C). Based on our previous work [53], we optimized the nanoelectroporation setup for the CRISPRi system, which requires larger plasmids, by determining voltage and nanosubstrate pore diameter to achieve optimal transfection efficiency while maintaining high cell viability. We then transfected INS1 β‐cells with the sgRNA and dCas9‐KRAB plasmids and investigated the effect on *Ins1* expression.

## 2 Results

### 2.1 Nanopore Substrates With Pore Diameters of 200 nm and 28 V Applied Voltage Identified as Optimal Parameters for CRISPRi System Delivery

In the present study, we used a combination of two plasmids, one 3.5 kbp plasmid coding for a sgRNA targeting a sequence near the transcription start site (TSS) of the rat insulin promoter and one 8 kbp plasmid coding for dCas9‐KRAB (Figures S1 and S2), to downregulate *Ins1* in INS1 β‐cells by CRISPRi. Since the dCas9‐KRAB plasmid is considerably larger than the plasmids used in our previous work [53], we first performed a voltage‐ and pore‐diameter optimization to achieve a high transfection efficiency while maintaining high cell viability. According to our previous paper [53], using nanopores or nanostraws for transfecting the GFP‐coding plasmid pMAX to INS1 β‐cells had different effects on transfection efficiency and cell viability. Nanopores were gentler than nanostraws and resulted in a higher cell count after 48 h, which is essential for the application in this study where the transfected plasmids need time to yield an effect on insulin gene expression. We thus settled on using nanopores. Previously, we also found that nanopore surface chemistry played a significant role in transfection efficiency [54], where an alumina surface gave the best results. Hence, the nanopores used in this study have an alumina‐coated surface.

**FIGURE 2:**
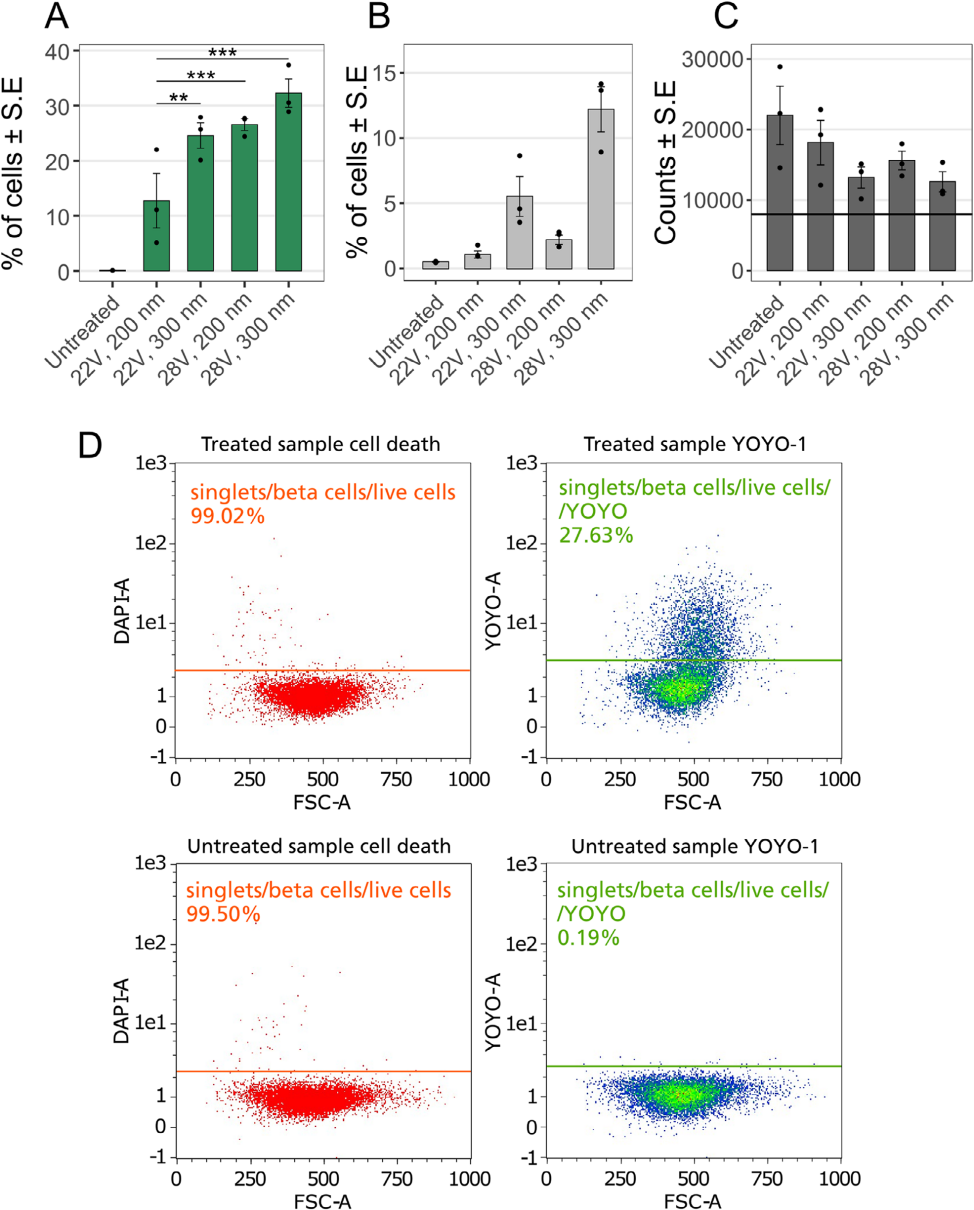
Nanopore diameter and voltage optimization of transfection efficiency and cell viability immediately after transfection with the dCas9‐KRAB plasmid stained with YOYO‐1. Column scatter plot displays of (A) Percentage of live cells transfected (Mean value ± S.E.), (B) Percentage of dead cells (Mean value ± S.E.), (C) Number of cells 48 h after transfection when seeding 8000 cells from each sample, indicated by the black horizontal line (Mean value ± S.E.). The given pore diameters are the nominal pore diameters from the membrane manufacturer, before deposition of alumina. *n* = 3, ^***^ *p* < 0.001, ^**^ *p* < 0.01, ANOVA and Tukey Post Hoc test. (D) Gating for cell death and transfection, shown for one of the 28 V, 200 nm experiments. The *x*‐axis of all panels is the area of the forward scatter signal, the *y*‐axis is the area of the DAPI fluorescence intensity signal (DAPI‐A), and the area of the YOYO‐1 fluorescence intensity signal (YOYO‐A). For the full gating strategy, see Figure S3.

The pore diameters displayed in Figure 2A,B are the nominal pore diameters given by the manufacturer, that is, before alumina coating. For optimization of the pore diameter and voltage, we used the dCas9‐KRAB plasmid, the larger of the two plasmids. The Cas9‐KRAB plasmid was stained with the DNA intercalating dye YOYO‐1 to enable immediate flow cytometry analysis of fluorescence after transfection (see gating in Figure 2C). Using nanopore substrates with 200 nm pore diameter led to lower cell death immediately after transfection (Figure 2A) and to a higher cell count after 48 h (Figure 2B) compared to using 300 nm pores. Applying pulses with an amplitude of 22 V, together with the 200 nm diameter pores, resulted in a slightly higher cell count than when applying 28 V pulses. However, there was a significantly higher percentage of transfected β‐cells with 28 V pulses, therefore 200 nm pore membranes and 28 V were selected.

### 2.2 Successful Delivery of CRISPRi System into INS1 β‐cells Using Nanopore Electroporation

**FIGURE 3:**
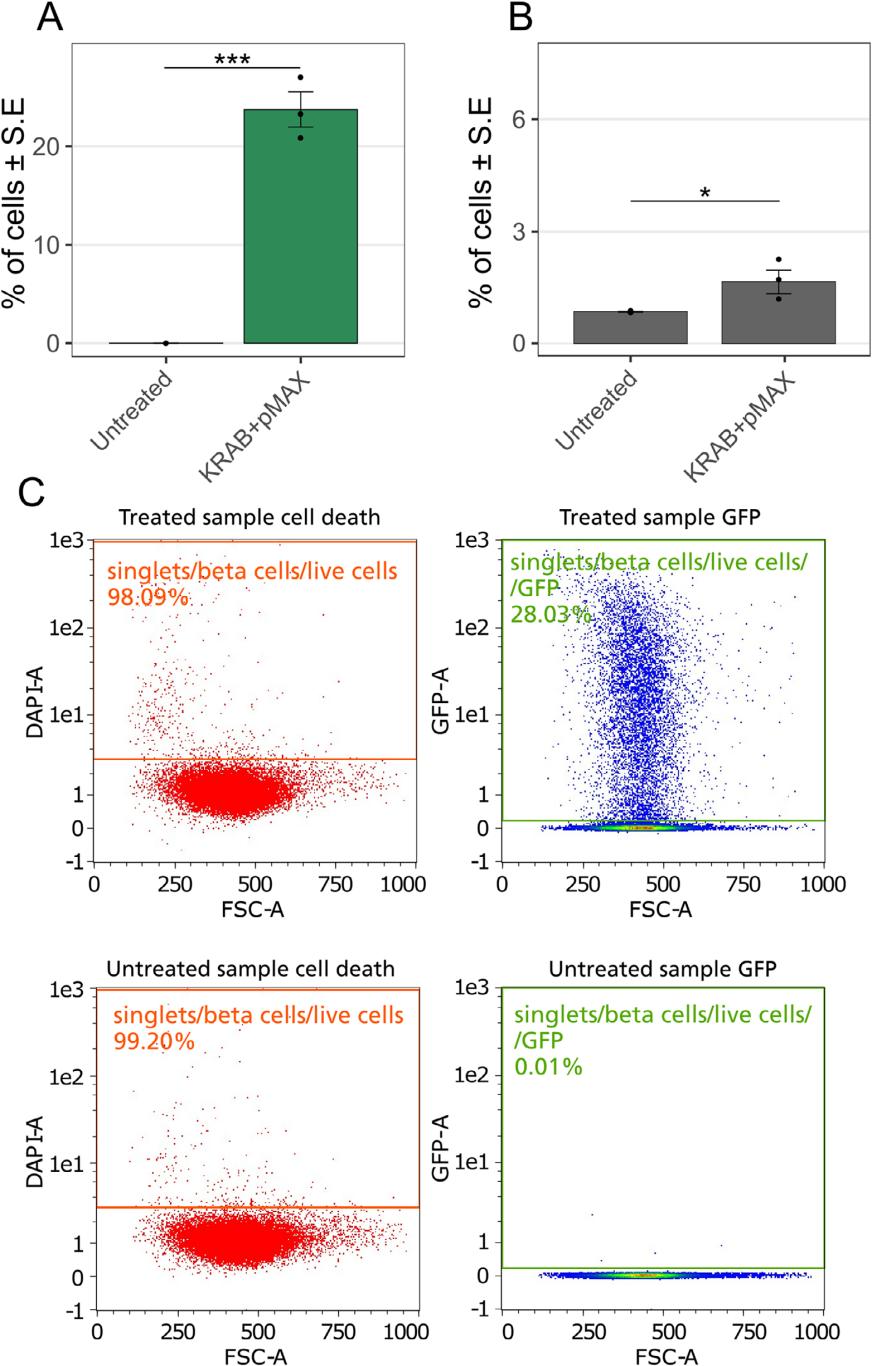
GFP expression 48 h after transfection of dCas9‐KRAB and pMAX plasmids. The GFP‐reporter is located on the pMAX plasmid, which is of the same size as the sgRNA plasmid that is injected to achieve a downregulation of insulin. Column scatter plot displays of (A) Percentage of live cells transfected (Mean value ± S.E.) and (B) Percentage of dead cells (Mean value ± S.E.). *n* = 4, ^***^ *p* < 0.001, ^*^ *p* < 0.05, *t*‐test. (C) Gating for cell death and GFP expression after transfection shown for one of the experiments. The *x*‐axis of all panels is the area of the forward scatter signal, the y‐axis is the area of the DAPI fluorescence intensity signal (DAPI‐A) and the area of the GFP fluorescence intensity signal (GFP‐A). For the full gating strategy, see Figure S4.

To evaluate whether the plasmids stay in the INS1 β‐cells long enough to become transcribed and translated, GFP expression was analyzed 48 h after transfection with the dCas9‐KRAB plasmid together with the GFP‐coding plasmid pMAX, which is of similar size as the sgRNA plasmid. When injecting these two plasmids together, about 24% of the INS1 β‐cells were positive for GFP after 48 h, which means that 24% of the INS1 β‐cells received, transcribed and translated the pMAX plasmid (Figure 3). The reason for injecting both plasmids simultaneously was to mimic the size of the plasmids included in the CRISPRi system, dCas9‐KRAB+sgRNA plasmids, that will be used for downregulating insulin. The sgRNA plasmid was exchanged for the pMAX plasmid to have a reliable readout method with flow cytometry, analyzing the GFP signal.

### 2.3 Downregulation of *Ins1* in INS1 β‐cells Using Nanopore Electroporation

**FIGURE 4:**
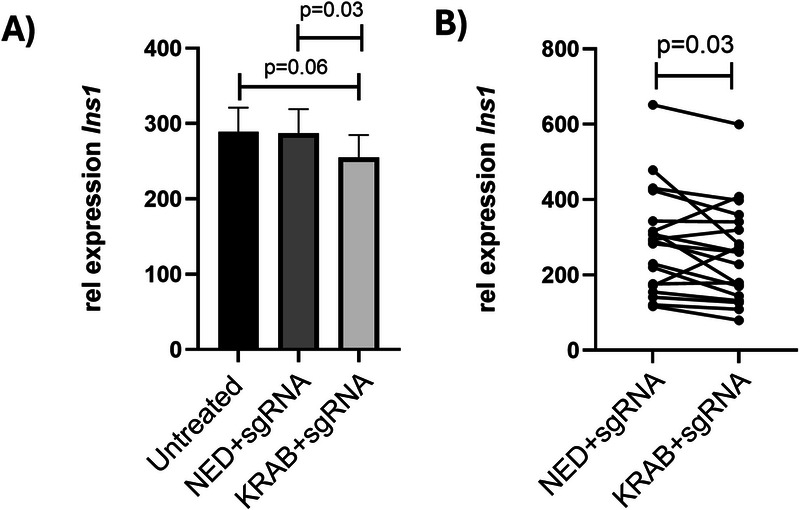
Relative *Ins1* gene expression based on qPCR analysis of clonal β‐cells transfected with dCas9‐NED and sgRNA (NED+sgRNA), or dCas9‐KRAB and sgRNA (KRAB+sgRNA) using nanopore electroporation. The relative gene expression was determined by averaging the triplicates within each experiment before using the $2^{-\Delta \Delta C_{T}}$ method [55]. *Hprt1* was used as an endogenous control. (A) Column graph display of the mean relative *Ins1* gene expression for all experiments (mean value ± S.E.), (*n* = 19). (B) Line graph of paired NED and KRAB results for each experiment. (The statistics were calculated with a Wilcoxon matched pairs signed rank test; *p*‐value: two‐tailed).

The INS1 β‐cells were then transfected with the CRISPRi system, the combination of dCas9‐KRAB and sgRNA plasmids, to downregulate *Ins1* gene expression. Two controls were used: (i) untreated cells, and (ii) the effector backbone plasmid (dCas9‐No Effector Domain (NED)) together with the sgRNA plasmid. The latter is mimicking dCas9‐KRAB+sgRNA plasmids in all aspects, except that the dCas9‐NED plasmid does not encode the transcription regulator. Figure 4A presents the mean relative expression of all experiments (*n* = 19, each performed in triplicates), analyzed byquantitative polymerase chain reaction (qPCR), and shows a significant down‐regulation of *Ins1* gene expression when comparing INS1 β‐cells transfected with dCas9‐KRAB+sgRNA and INS1 β‐cells that had received the dCas9‐NED+sgRNA (*p* = 0.03). Further we also detected a trend (*p* = 0.06) for lower *Ins1* gene expression in INS1 β‐cells that had received the dCas9‐KRAB plasmid + sgRNA plasmid compared to the untreated INS1 β‐cells. In Figure 4B, dCas9‐NED+sgRNA and dCas9‐KRAB+sgRNA are paired for each respective experiment, showing that in most of the experiments (all but four), there is a decrease in the relative expression of *Ins1* for CRISPRi versus the control. Together, these results demonstrate that nanoelectroporation successfully enabled CRISPRi‐mediated downregulation of *Ins1* in INS1 β‐cells.

## 3 Discussion

In this study, nanopore electroporation was used to transfect INS1 β‐cells with the CRISPRi system, resulting in successful downregulation of *Ins1*. This study shows the feasibility of using nanopore electroporation for the transfection of large constructs needed for epigenetic editing into a hard to transfect cell type, thus paving the way for epigenetic editing in the analysis of the causative roles of epigenetic changes in disease development.

Two plasmids of significantly different sizes, sgRNA and dCas9‐KRAB were injected inside the cells, and since the dCas9‐KRAB plasmid is considerably larger than the plasmids we have used until here, optimization of some transfection parameters was first required, where four combinations of two voltages and two pore diameters were tested. Using small pores with a low voltage resulted in the lowest transfection efficiency, probably owing to insufficient electrophoretic force for the big plasmid to be pulled through the nanopores. For a given voltage, the 200 nm pores resulted in a lower percentage of transfected cells but higher cell survival and higher cell count compared to when using 300 nm pores. Since the porosities of the 200 and 300 nm pore substrates are the same, the electrical resistance of both substrates and the electrical field in the pores are similar. However, the size of the pores in itself can influence both efficiency and cell survival. Indeed, on one hand, larger pores will make it easier to deliver plasmids because of lower mechanical hindrance. On the other hand, larger pores lead to more challenging cell membrane recovery after nanoelectroporation which can lead to cell death.

In general, all treated samples had a lower cell count 48 h after transfection compared to controls, which was caused either by the nanopore electroporation per se, or by a cytotoxic effect of the plasmids. In a previous study, we have ruled out proliferation as a possible factor behind the lower cell count. Instead, we have observed cell detachment from the substrate after transfection and reseeding [54]. The results suggested a positive correlation between the amount of plasmid delivered in the cells and cell detachment, raising the issue of plasmid cytotoxicity. This is supported by the fact that nanoelectroporation methods have on several occasions been established to be minimally invasive and maintain high cell viability [42, 56], while large plasmids have been found to be cytotoxic [57]. The plasmid cytotoxicity could also possibly contribute to the lower viability of cells transfected with larger pore diameter, as larger pores could enable easier transport of the large plasmid.

A drawback of our experimental setup is that we cannot use flow cytometry to evaluate how many cells are transfected with both plasmids. This is because the dCas9‐KRAB plasmid has no fluorescent reporter, and that staining the two plasmids with different intercalating dyes would be unreliable since the dyes can bind to both plasmids indiscriminately when mixed. Instead, we replaced the sgRNA plasmid with a GFP reporter plasmid. This showed that GFP was expressed by 25% of the cells. Since the dCas9‐KRAB plasmid has no fluorescent reporter, the transfection of the dCas9‐KRAB plasmid in these cells is unknown. However, it is unlikely that cells would be transfected with only one plasmid as shown by a similar electroporation system using nanostraws instead of nanopores [43]. Additionally, another study showed an increased delivery of large plasmids when a smaller plasmid was used simultaneously [58]. Since we observed a 25% immediate transfection efficiency for the dCas9‐KRAB plasmid and 25% of cells expressing GFP after co‐transfection with dCas9‐KRAB and pMAX plasmids, we speculate that roughly 25% of the cells may be successfully affected by the CRISPRi system in our experiments. A 25% efficiency is lower than what was reported in other studies where efficiencies around 80% for GFP plasmids, large proteins [38, 47, 48] and GFP RNA [39] have been reported. We believe that it is due to a combination of the hard‐to‐transfect nature of the clonal β‐cells and the large size of the dCas9‐KRAB plasmid compared to the cargos used in these studies. In our previous study, when transfecting clonal β‐cells with a 3.5 kbp GFP plasmid, we obtained an efficiency of 50%–60% [54]. With the larger dCas9‐KRAB plasmid (8 kbp, ≥ 100 nm in diameter [59]), we can expect a much lower delivery efficiency. For comparison, reported genetic editing efficiencies when transfecting cells with CRISPR‐Cas9 ribonucleoproteins (RNPs) using nanoelectroporation are between 25% and 35% [38, 39, 48]. Note that the size of the dCas9‐KRAB plasmid is substantially larger than RNPs, which are around 12 nm in diameter. Taken together, the size factor, combined with the hard to transfect character of the clonal β‐cells can explain the 25% efficiency.

To our knowledge, nanoelectroporation has never been used for the modification of gene expression with the CRISPRi system. We show a significant downregulation of *Ins‐1* expression after CRISPRi transfection. This shows that the sgRNA targeted the correct site and that KRAB had the intended effect on *Ins1* expression and thereby confirmed that the CRISPRi system worked. The difference in mean relative expression (Figure 4) is rather subtle. However, ≈ 25% of the cells are transfected and therefore most cells will express normal amounts of insulin mRNA, indicating that the effect in reality is much more pronounced. It has also been found that the sgRNA position in the genome is an important factor when using the CRISPRi and CRISPRoff systems [60, 61], so testing sgRNAs targeting other loci could possibly lead to a stronger downregulation. Moreover, as insulin is extremely abundant in β‐cells [62], it may be difficult to see strong effects of CRISPRi on the expression of this gene. Because of this, one may consider also studying genes expressed at lower levels and evaluate whether the relative effects of CRISPRi would become stronger. Future experiments may also evaluate nanopore electroporation of CRISPRi in other cell types and the regulation of other genes. Whereas the CRISPRi used in the present study utilizes a transcription factor to downregulate gene expression, future studies may also test whether nanopore electroporation could be used for epigenetic editing using dCas9 combined with e.g., DNMTs or TETs, resulting in altered DNA methylation of the locus targeted by the sgRNA [27, 28]. In conclusion, this study opens new possibilities for causal studies of epigenetic mechanisms, by providing a broadly applicable, non‐viral delivery strategy for the CRISPR‐dCas9 system in difficult‐to‐transfect cells.

## 4 Materials and Methods

### 4.1 Nanopore Fabrication and Nanopore Electroporation Device Assembly

Track‐etched polycarbonate (PC) membranes (it4ip, Louvain‐la‐Neuve, Belgium) were used as a base substrate for nanopore fabrication. In the track‐etching process, a thin polymer film is first irradiated with energetic ions, creating linear damage tracks. These tracks are more chemically reactive than the surrounding polymer and can be selectively etched into pores, with pore size and shape determined by the etching conditions. The nanopore membranes were coated with alumina using atomic layer deposition (ALD, Savannah, Cambridge Nanotech, Cambridge, MA, USA). To achieve an approximately 12 nm thick alumina layer on the PC membrane, 130 cycles of alternating 0.15 s trimethylaluminum (TMAl) and water pulses, with 30 s inter‐pulse waiting time, were employed at 90°C. The PC membranes were 25 µm thick and had a nominal pore diameter of 200 or 300 nm. Membranes of both pore diameters had a porosity of ∼0.6%, corresponding to 2E7 pores/cm^2^ for the 200 nm pores and 9E6 pores/cm^2^ for the 300 nm pores.

Nanopore electroporation devices consisted of the nanopore substrate adhered to a plastic cylinder (1 cm high and 4 mm in inner diameter) with double‐sided tape (3 8153LE (300LSE) double‐lined Adhesive Transfer), creating a reservoir (see Figure 1A).

### 4.2 Cell Culture

The insulin‐producing INS1 β‐cells (832/13 INS‐1) were used for this work and kept within a population doubling number of 21–60 [63]. The INS1 β‐cells were cultured in RPMI‐1640 medium supplemented with 10% heat‐inactivated fetal bovine serum (FBS, qualified, Brazil origin, Gibco, ThermoFisher Scientific, Waltham, MA, USA), 1% penicillin‐streptomycin (PenStrep, Sigma Aldrich, Burlington, MA, USA), 2.2% supplement consisting of 50% sodium pyruvate solution 100 mm (Gibco, ThermoFisher Scientific), 50% glutamine solution 200 mm (Gibco, ThermoFisher Scientific), and 176 ppm 2‐mercaptoethanol. This will hereafter be referred to as complete medium. Cells were kept at 37°C with 5% CO_2_ and passaged when reaching 80%–100% confluency. For passaging, the INS1 β‐cells were washed with 1×Dulbecco's phosphate‐buffered saline (DPBS, VWR, Radnor, PA, USA) before incubation with trypsin‐EDTA (Gibco, ThermoFisher Scientific) for 3 min and resuspension in complete medium. The suspension was then centrifuged for 3 min at 700 g before the pellet was re‐suspended in complete medium, and about 25% of the clonal β‐cells could be re‐seeded in a new culture flask.

### 4.3 Plasmids

Four different plasmids were used in this work: (i) coding for the sgRNA (Addgene MLM3636 #43860; MLM3636 was a gift from Keith Joung (Addgene plasmid # 43860; http://n2t.net/addgene:43860; RRID: Addgene_43860))(Figure S1), (ii) coding for dCas9 with the artificial transcription factor KRAB (Figure S2), (iii) coding for dCas9 but no Effector Domain NED (Figure S5)(Addgene pMLM3705 # 47754) [52, 64], and (iv) the pMAX plasmid (GFP‐encoding, prepared by the Cell & Gene Therapy Core at Lund Stem Cell Center) (Figure S6). The sgRNA matches a DNA sequence located near the transcription start site of the *Ins1* gene (see below) and guides dCas9 to that location. The dCas9 will then bind to the DNA, and the effector domain KRAB connected to the dCas9 will downregulate transcription of *Ins1*. The NED plasmid is used as a control, as it provides an identical system to the one with KRAB but without being able to modify the gene expression levels.

### 4.4 sgRNA Design and Sequence

The sgRNA was designed using CRISPOR [65]. The sgRNA has the sequence CTTTGCCGTTTGGCCCATTA AGG and is targeting the rat *Ins1* gene close to the TSS. The following primer pair was used to clone the sgRNA into the MLM3636 vector (Plasmid #43860, Addgene, Watertown, MA, USA) as previously described (Primer sense (Sequence 5′‐3′): ACACCGCTTTGCCGTTTGGCCCATTAG and Primer antisense (Sequence 5′‐3′): AAAACTAATGGGCCAAACGGCAAAGCG) [52].

### 4.5 Nanopore Electroporation of INS1 β‐cells

After resuspending INS1 β‐cells according to the passaging protocol, the cell suspension was diluted 10 times in DPBS containing DAPI (0.01 µg/ml final concentration) before determination of cell concentration and viability using flow cytometry on a MACSQuant Analyzer 16 Flow cytometer with MACSQuant running buffer, storage solution, and washing solution (Miltenyi Biotech, Bergisch Gladbach, Germany).

#### 4.5.1 Cargos

For the optimization of nanopore electroporation, the dCas9‐KRAB plasmid (0.2 µg/µl in MilliQ water) was stained with the intercalating dye YOYO‐1 iodide (Invitrogen, ThermoFisher). The YOYO‐1 dye 1 mm stock solution was diluted to 100 µm in MilliQ water and added to the plasmid solution to a final ratio of 1:250 (YOYO‐1 molecules: base pairs), after which the solution was kept at 50°C for 2 h. To further evaluate whether the plasmids were transcribed, the KRAB and pMAX plasmids were transfected simultaneously at a total concentration of 400 ng/µl (200 ng/µl of each plasmid type). Here, pMAX was used instead of the sgRNA plasmid because of its GFP reporter enabling easy analysis. When transfecting sgRNA+dCas9‐KRAB or sgRNA+dCas9‐NED, a mixture of the sgRNA plasmid and dCas9 plasmid in MQ water was used as a cargo solution. The total DNA concentration was 400 ng/µl, with 200 ng/µl of each plasmid type.

For nanopore electroporation experiments, 35 000 INS1 β‐cells were seeded in 100 µl complete cell medium in a device. The devices were subsequently placed in a 24‐well plate with complete medium surrounding them and then centrifuged for 1 min at 200 g to promote a good seal between INS1 β‐cells and nanopore substrate. Just before performing electroporation, 70 µL of cell medium was removed from each device.

### 4.6 Nanopore Electroporation Process

The nanopore electroporation setup consisted of two electrodes, a gold‐coated glass slide (100 nm Au thickness, Platypus Technologies, Fitchburg, WI, USA) and a platinum wire (0.5 mm in diameter), a signal generator (TGP110, Aim and Thurlby Thandar Instruments, Huntingdon, UK) and an amplifier (WMA‐300, Falco Systems BV, Katwijk aan Zee, Netherlands) for generating the electric pulses, as well as an oscilloscope for observing the signal. The device was placed on top of a 15 µL drop of plasmid solution deposited on the gold electrode, after which the top platinum electrode was inserted into the cell medium in the device, with an inter‐electrode distance of 0.5 mm. A train of square electrical pulses (200 µs wide, 28 V, 40 Hz) was then applied for 40 s, after which the device was lifted and put back down again before applying another train of pulses for 40 s. After treatment, the device was placed in a drop of cell medium to rinse the plasmid solution below the nanopore substrate. After cells from triplicate devices had been nanoelectroporated, they were resuspended by gently pipetting up and down to be analyzed as described below.

For the voltage‐ and pore diameter optimization experiments, half the volume of the cell solution for each sample was analyzed using flow cytometry to determine the immediate transfection efficiency (YOYO‐1 fluorescence), cell viability (DAPI fluorescence; DAPI was added to the cell solution before analysis at a final concentration of 0.01 ug mL−1), and cell concentration. Subsequently, 8000 cells per sample were seeded in a 48‐well plate and cultured for 48 h before being resuspended by trypsinization (by rinsing cells with DPBS and adding 50 µL trypsin for 3 min, after which 250 µL of complete medium was added). The cell count was then assessed using flow cytometry. Three of these experiments were conducted, each with triplicates of each sample type.

For the experiments assessing the GFP‐expression and the effect on *Ins1* gene expression, after nanopore electroporation, cells were resuspended by pipetting up and down and then directly re‐seeded in a 48‐well plate and cultured for 48 h. The medium was changed after 24 h. After 48 h, the cells were resuspended by rinsing with DPBS and trypsinized as described above. For the experiments where GFP was expressed, the cell GFP fluorescence was then analyzed using flow cytometry. Three GFP‐expression experiments, in triplicates, were done. For the gene expression experiment, the cell solution was transferred to Eppendorf tubes before centrifugation for 5 min at 300 g. The supernatant was removed, and the pellets were frozen at −80°C until analyzed using qPCR. For this, 19 independent experiments were conducted, with triplicates in each.

### 4.7 RNA Extraction, cDNA Preparation and qPCR

Total RNA was extracted from nanopore‐transfected INS1 β‐cells with the RNeasy Micro kit (Qiagen, Venlo, Netherlands) and converted to complementary DNA (cDNA) with the RevertAid First Strand cDNA synthesis kit (ThermoFisher Scientific). Quantitative PCR (qPCR) was performed in triplicates in 384‐well plates and Applied Biosystems QuantStudio 7 Flex Real‐Time PCR system (ThermoFisher Scientific) under default cycling parameters. TaqMan assays were used to measure the mRNA expression of rat *Ins1* (Rn02121433_g1) and rat *Hprt1* (RN01527840_m1), where the latter was the endogenous control used to normalize *Ins1* mRNA expression levels. TaqMan assays and qPCR reagents were purchased from ThermoFisher Scientific. Relative expression was calculated with the $2^{-\Delta \Delta C_{T}}$ method [55].

### 4.8 Statistical Analysis

For the injected KRAB‐plasmid stained with YOYO‐1, the percentage of cells and cell count were directly extracted from the flow cytometry software for each sample. The mean value of each triplicate was calculated, and the triplicates from all experiments (*n* = 3) were then averaged to be displayed as bar graphs with standard error (S.E.). The statistical significance was assessed using a one‐way ANOVA with a Tukey post hoc test (^***^p < 0.001, ^**^p<0.01, ^*^p<0.05). For the GFP expression experiments, a t‐test (^***^p < 0.001, ^**^p<0.01, ^*^p<0.05) was performed (*n* = 4). The statistical analysis of these results was conducted in R. For qPCR analysis, the data are reported as relative expression and normalized to an endogenous control (*Hprt1* see above). Relative expression was calculated with the $2^{-\Delta \Delta C_{T}}$ method [55]. qPCR data are displayed as the mean ± S.E., and in total 19 sample pairs were evaluated (*n* = 19). GraphPad software was used to perform statistical analysis and to assess statistical significance of the qPCR data, and a Wilcoxon matched pairs signed rank test (non‐parametric) was applied.

## Conflicts of Interest

The authors declare no conflicts of interest.

## Supporting information




**Supporting File**: smll73191‐sup‐0001‐SuppMat.docx.

## Data Availability

The data that support the findings of this study are available from the corresponding author upon reasonable request.

## References

[1] International Diabetes Federation , IDF Diabetes Atlas 10th Edition, 10th ed. (International Diabetes Federation, 2021).

[2] P. A. Halban , K. S. Polonsky , D. W. Bowden , et al., “β‐Cell Failure in Type 2 Diabetes: Postulated Mechanisms and Prospects for Prevention and Treatment,” Diabetes Care 37 (2014): 1751–1758, 10.2337/dc14-0396. 24812433 PMC4179518

[3] K. Bacos , A. Perfilyev , A. Karagiannopoulos , et al., “Type 2 Diabetes Candidate Genes, Including PAX5, Cause Impaired Insulin Secretion in Human Pancreatic Islets,” Journal of Clinical Investigation 133 (2023): 163612. 10.1172/JCI163612 PMC9927941 36656641

[4] G. Wang , J. Chiou , C. Zeng , et al., “Integrating Genetics With Single‐cell Multiomic Measurements Across Disease States Identifies Mechanisms of Beta Cell Dysfunction in Type 2 Diabetes,” Nature Genetics 55 (2023): 984–994. 37231096 10.1038/s41588-023-01397-9 PMC10550816

[5] C. Ling , K. Bacos , and T. Rönn , “Epigenetics of Type 2 Diabetes Mellitus and Weight Change — A Tool for Precision Medicine?,” Nature Reviews Endocrinology 18 (2022): 433–448, 10.1038/s41574-022-00671-w. 35513492

[6] C. Ling and L. Groop , “Epigenetics: A Molecular Link Between Environmental Factors and Type 2 Diabetes,” Diabetes 58 (2009): 2718–2725. 19940235 10.2337/db09-1003 PMC2780862

[7] R. A. Kowluru and G. Mohammad , “Epigenetic Modifications in Diabetes,” Metabolism 126 (2022): 154920. 34715117 10.1016/j.metabol.2021.154920 PMC10277168

[8] C. Ling and T. Rönn , “Epigenetics in Human Obesity and Type 2 Diabetes,” Cell Metabolism 29 (2019): 1028–1044. 30982733 10.1016/j.cmet.2019.03.009 PMC6509280

[9] C. Davegårdh , J. Säll , A. Benrick , et al., “VPS39‐deficiency Observed in Type 2 Diabetes Impairs Muscle Stem Cell Differentiation via Altered Autophagy and Epigenetics,” Nature Communications 12 (2021): 2431, 10.1038/s41467-021-22068-5. PMC8065135 33893273

[10] E. Nilsson , A. Matte , A. Perfilyev , et al., “Epigenetic Alterations in Human Liver from Subjects With Type 2 Diabetes in Parallel With Reduced Folate Levels,” The Journal of Clinical Endocrinology & Metabolism 100 (2015): E1491–E1501, 10.1210/jc.2015-3204. 26418287 PMC4702449

[11] E. Nilsson , M. Vavakova , A. Perfilyev , et al., “Differential DNA Methylation and Expression of miRNAs in Adipose Tissue From Twin Pairs Discordant for Type 2 Diabetes,” Diabetes 70 (2021): 2402–2418. 34315727 10.2337/db20-0324

[12] E. Nilsson , P. A. Jansson , A. Perfilyev , et al., “Altered DNA Methylation and Differential Expression of Genes Influencing Metabolism and Inflammation in Adipose Tissue From Subjects With Type 2 Diabetes,” Diabetes 63 (2014): 2962–2976, 10.2337/db13-1459. 24812430

[13] T. Rönn , J. K. Ofori , A. Perfilyev , et al., “Genes With Epigenetic Alterations in human Pancreatic Islets Impact Mitochondrial Function, Insulin Secretion, and Type 2 Diabetes,” Nature Communications 14 (2023): 1–21. 10.1038/s41467-023-43719-9 PMC10716521 38086799

[14] P. Volkov , K. Bacos , J. K. Ofori , et al., “Whole‐Genome Bisulfite Sequencing of human Pancreatic Islets Reveals Novel Differentially Methylated Regions in Type 2 Diabetes Pathogenesis,” Diabetes 66 (2017): 1074–1085, 10.2337/db16-0996. 28052964

[15] T. Dayeh , P. Volkov , C. Bacos , et al., “Genome‐Wide DNA Methylation Analysis of Human Pancreatic Islets From Type 2 Diabetic and Non‐Diabetic Donors Identifies Candidate Genes That Influence Insulin Secretion,” PLos Genet 10 (2014): 1004160. 10.1371/journal.pgen.1004160 PMC3945174 24603685

[16] T. Rönn , A. Perfilyev , N. Oskolkov , and C. Ling , “Predicting Type 2 Diabetes via Machine Learning Integration of Multiple Omics From Human Pancreatic Islets,” Scientific Reports 14 (2024): 14637. 38918439 10.1038/s41598-024-64846-3 PMC11199577

[17] C. Ling , M. Vavakova , B. Ahmad Mir , et al., “Multiomics Profiling of DNA Methylation, microRNA, and mRNA in Skeletal Muscle From Monozygotic Twin Pairs Discordant for Type 2 Diabetes Identifies Dysregulated Genes Controlling Metabolism,” BMC Medicine 22 (2024): 572, 10.1186/s12916-024-03789-y. 39623445 PMC11613913

[18] E. Hall , T. Dayeh , C. L. Kirkpatrick , C. B. Wollheim , M. Dekker Nitert , and C. Ling , “DNA Methylation of the Glucagon‐Like Peptide 1 Receptor (GLP1R) in human Pancreatic Islets,” BMC Medical Genetics 14 (2013): 76, 10.1186/1471-2350-14-76. 23879380 PMC3727960

[19] B. T. Yang , K. Bacos , T. Rönn , C. Ling , and L. Groop , “Increased DNA Methylation and Decreased Expression of PDX‐1 in Pancreatic Islets From Patients With Type 2 Diabetes,” Molecular Endocrinology 26 (2012): 1203–1212. 22570331 10.1210/me.2012-1004 PMC5416998

[20] A. H. Olsson , T. Rönn , C. Ling , and L. Groop , “Genome‐Wide Associations Between Genetic and Epigenetic Variation Influence mRNA Expression and Insulin Secretion in Human Pancreatic Islets,” PLos Genet 10 (2014): 1004735 . 10.1371/journal.pgen.1004735 PMC4222689 25375650

[21] K. Bacos , L. Gillberg , P. Volkov , et al., “Blood‐based Biomarkers of Age‐Associated Epigenetic Changes in Human Islets Associate With Insulin Secretion and Diabetes,” Nature Communications 7 (2016): 11089, 10.1038/ncomms11089. PMC4821875 27029739

[22] S. R. McCutcheon , D. Rohm , N. Iglesias , and C. A. Gersbach , “Epigenome Editing Technologies for Discovery and Medicine,” Nature Biotechnology 42 (2024): 1199–1217, 10.1038/s41587-024-02320-1. 39075148

[23] X. S. Liu , H. Wu , X. Ji , et al., “Editing DNA Methylation in the Mammalian Genome,” Cell 167 (2016): 233–247.e17, 10.1016/j.cell.2016.08.056. 27662091 PMC5062609

[24] E. A. Heller , L. Bintu , and M. G. Rots , “Epigenetic Editing: from Concept to Clinic,” Nature Reviews Drug Discovery 25 (2025): 227–248. 41286458 10.1038/s41573-025-01323-0

[25] J. H. Goell and I. B. Hilton , “CRISPR/Cas‐Based Epigenome Editing: Advances, Applications, and Clinical Utility,” Trends in Biotechnology 39 (2021): p678–691. 10.1016/j.tibtech.2020.10.012 33972106

[26] M. Nakamura , Y. Gao , A. A. Dominguez , and L. S. Qi , “CRISPR Technologies for Precise Epigenome Editing,” Nature Cell Biology 23 (2021): 11–22. 33420494 10.1038/s41556-020-00620-7

[27] A. Vojta , J. Dobrinic , T. Tadic , and I. Bencic , “Repurposing the CRISPR‐Cas9 System for Targeted DNA Methylation,” Nucleic Acids Research 44 (2016): 5615–5628. 26969735 10.1093/nar/gkw159 PMC4937303

[28] S. R. Choudhury , Y. Cui , K. Lubecka , B. Stefanska , and J. Irudayaraj , “CRISPR‐dCas9 Mediated TET1 Targeting for Selective DNA Demethylation at BRCA1 Promoter,” Oncotarget 7 (2016): 46545–46556. 27356740 10.18632/oncotarget.10234 PMC5216816

[29] R. A. F. Gjaltema , D. Goubert , C. Huisman , et al., “KRAB‐induced Heterochromatin Effectively Silences PLOD2 Gene Expression in Somatic Cells and Is Resilient to tgfβ1 Activation,” International Journal of Molecular Sciences 21 (2020): 3634, 10.3390/ijms21103634. 32455614 PMC7279273

[30] F. Sarno , M. D. Silva , L. G. Souza , and A. R. Almeida , “Epigenetic Editing and Epi‐drugs: A Combination Strategy to Simultaneously Target KDM4 as a Novel Anticancer Approach,” Clin Epigenetics 17 (2025): 105. 40537846 10.1186/s13148-025-01913-0 PMC12177974

[31] A. C. H. van den Berg van Saparoea , T. K. Van Dijk , and R. F. Rots , “Plasmid Delivery and Single‐Cell Plasmid Expression Analysis for CRISPR/dCas9‐Based Epigenetic Editing,” Methods in Molecular Biology. (Springer, 2024). 10.1007/978-1-0716-4051-7_13 39012600

[32] M. Morshed , L. J. Yang , and H. K. Choi , “Non‐viral Delivery Systems of DNA Into Stem Cells: Promising and Multifarious Actions for Regenerative Medicine,” Journal of Drug Delivery Science and Technology 60 (2020): 101861.

[33] D. Pei and M. Buyanova , “Overcoming Endosomal Entrapment in Drug Delivery,” Bioconjugate Chemistry 30 (2019): 273–283, 10.1021/acs.bioconjchem.8b00778. 30525488 PMC6501178

[34] T. K. Kim and J. H. Eberwine , “Mammalian Cell Transfection: The Present and the Future,” Analytical and Bioanalytical Chemistry 397 (2010): 3173–3178. 20549496 10.1007/s00216-010-3821-6 PMC2911531

[35] N.‐B. Woods , “Lentiviral Vector Transduction of NOD/SCID Repopulating Cells Results in Multiple Vector Integrations per Transduced Cell: Risk of Insertional Mutagenesis,” Blood 101 (2003): 1284–1289, 10.1182/blood-2002-07-2238. 12393514

[36] X. Du , J. Wang , Q. Zhou , et al., “Advanced Physical Techniques for Gene Delivery Based on Membrane Perforation,” Drug Delivery 25 (2018): 1516–1525, 10.1080/10717544.2018.1480674. 29968512 PMC6058615

[37] A. K. Fajrial , Q. Q. He , N. I. Wirusanti , J. E. Slansky , and X. Ding , “A Review of Emerging Physical Transfection Methods for CRISPR/Cas9‐Mediated Gene Editing,” Theranostics 10 (2020): 5532–5549. 32373229 10.7150/thno.43465 PMC7196308

[38] Y. Cao , E. Ma , S. Cestellos‐Blanco , et al., “Nontoxic Nanopore Electroporation for Effective Intracellular Delivery of Biological Macromolecules,” Proceedings of the National Academy of Sciences U S A 116 (2019): 7899–7904, 10.1073/pnas.1818553116. PMC6475394 30923112

[39] Y. Cao , H. Chen , R. Qiu , et al., “Universal Intracellular Biomolecule Delivery With Precise Dosage Control,” Science Advances 4 (2018): aat8131, 10.1126/sciadv.aat8131. PMC6209385 30402539

[40] M. A. Pop and B. D. Almquist , “Controlled Delivery of MicroRNAs Into Primary Cells Using Nanostraw Technology,” Advanced NanoBiomed Research 1 (2021): 2000033. 10.1002/anbr.202000061 PMC7611046 34164629

[41] P. Mukherjee , S. S. P. Nathamgari , J. A. Kessler , and H. D. Espinosa , “Combined Numerical and Experimental Investigation of Localized Electroporation‐Based Cell Transfection and Sampling,” ACS Nano 12 (2018): 12118–12128, 10.1021/acsnano.8b05473. 30452236 PMC6535396

[42] G. He , H.‐J. Chen , D. Liu , et al., “Fabrication of Various Structures of Nanostraw Arrays and Their Applications in Gene Delivery,” Advanced Materials Interfaces 5 (2018): 1701535, 10.1002/admi.201701535.

[43] X. Xie , A. M. Xu , S. Leal‐Ortiz , Y. Cao , C. C. Garner , and N. A. Melosh , “Nanostraw–Electroporation System for Highly Efficient Intracellular Delivery and Transfection,” ACS Nano 7 (2013): 4351–4358, 10.1021/nn400874a. 23597131

[44] A. Zhang , J. Fang , J. Wang , X. Xie , H.‐J. Chen , and G. He , “Interrogation on the Cellular Nano‐Interface and Biosafety of Repeated Nano‐Electroporation by Nanostraw System,” Biosensors 12 (2022): 522, 10.3390/bios12070522. 35884325 PMC9313307

[45] L. Schmiderer , A. Subramaniam , K. Zemaitis , et al., “Efficient and Nontoxic Biomolecule Delivery to Primary Human Hematopoietic Stem Cells Using Nanostraws,” Proceedings of the National Academy of Sciences 117 (2020): 21267–21273, 10.1073/pnas.2001367117. PMC7474688 32817519

[46] P. Mukherjee , M. Koncz , and A. Kiss , “Single Cell Transcriptomics Reveals Reduced Stress Response in Stem Cells Manipulated Using Localized Electric Fields,” Materials Today Bio 19 (2023): 100611. 10.1016/j.mtbio.2023.100601 PMC10102005 37063248

[47] C. A. Patino , N. Pathak , and J. Li , “Multiplexed High‐Throughput Localized Electroporation Workflow With Deep Learning–based Analysis for Cell Engineering,” Science Advances 8 (2022): abn1983. 10.1126/sciadv.abn7637 PMC9307252 35867793

[48] N. Pathak , C. A. Patino , M. Li , et al., “Cellular Delivery of Large Functional Proteins and Protein‐Nucleic Acid Constructs via Localized Electroporation,” Nano Letters 23 (2023): 3653–3660. 36848135 10.1021/acs.nanolett.2c04374 PMC10433461

[49] D. Volpati , P. H. B. Aoki , T. B. Johansson , et al., “Monitoring the Intracellular Fate of Molecular Beacons: The Challenge of False Positive Signals,” Advanced NanoBiomed Research 4 (2024), 10.1002/anbr.202300147.

[50] S. A. Camacho , P. H. B. Aoki , F. Ekstrand , O. N. Oliveira , and C. N. Prinz , “Enhancing Photothermal Therapy Against Breast Cancer Cells by Modulating the End Point of Gold Shell‐Isolated Nanoparticles Using Nanostraw‐Assisted Injection,” ACS Appl Mater Interfaces 17 (2025): 27816–27828. 40299396 10.1021/acsami.5c00084 PMC12086757

[51] E. Hebisch , M. Hjort , D. Volpati , and C. N. Prinz , “Nanostraw‐Assisted Cellular Injection of Fluorescent Nanodiamonds via Direct Membrane Opening,” Small 17 (2021): 2006421. 10.1002/smll.202006421 33502091

[52] D. Goubert , M. Koncz , A. Kiss , and M. G. Rots , “Establishment of Cell Lines Stably Expressing dCas9‐fusions to Address Kinetics of Epigenetic Editing” Epigenome Editing: Methods and Protocols, (Springer, 2018). 10.1007/978-1-4939-7774-1_22 29524148

[53] F. Ekstrand , C. Ling , K. Bacos , and T. Rönn , “Achieving Efficient Clonal Beta Cells Transfection Using Nanostraw/Nanopore‐Assisted Electroporation,” RSC advances 14 (2024): 22244–22252. 39010923 10.1039/d4ra02791d PMC11247384

[54] F. Ekstrand , S. Davidsson Bencker , S. Ruhrmann , Y. Yang , C. Ling , and C. N. Prinz , “Plasmid‐induced Cytotoxicity Revealed by Nanopore and Nanostraw Electroporation,” Nanoscale 17 (2025): 22382–22393, 10.1039/D5NR02352A. 40948305 PMC12434679

[55] K. J. Livak , T. D. Schmittgen , K. J. Livak , and T. D. Schmittgen , “Analysis of Relative Gene Expression Data Using Real‐time Quantitative PCR and the 2(‐Delta Delta C(T)) Method,” Methods 25 (2001): 402–408. 11846609 10.1006/meth.2001.1262

[56] J. Liu , X. Wang , Y. Cao , et al., “Nanopore Electroporation Device for DNA Transfection Into Various Spreading and Nonadherent Cell Types,” ACS Appl Mater Interfaces 15 (2023): 50015–50033. 37853502 10.1021/acsami.3c10939

[57] L. L. Lesueur , L. M. Mir , and F. M. André , “Overcoming the Specific Toxicity of Large Plasmids Electrotransfer in Primary Cells in Vitro,” Molecular Therapy—Nucleic Acids 5 (2016): 291, 10.1038/mtna.2016.4. PMC5014460 27111417

[58] J. N. Søndergaard , K. Geng , C. Sommerauer , I. Atanasoai , X. Yin , and C. Kutter , “Successful Delivery of Large‐size CRISPR/Cas9 Vectors in Hard‐to‐transfect human Cells Using Small Plasmids,” Communications Biology 3 (2020): 319. 32561814 10.1038/s42003-020-1045-7 PMC7305135

[59] M. J. Molloy , V. S. Hall , S. I. Bailey , K. J. Griffin , J. Faulkner , and M. Uden , “Effective and Robust Plasmid Topology Analysis and the Subsequent Characterization of the Plasmid Isoforms Thereby Observed,” Nucleic Acids Research 32 (2004): 129. 10.1093/nar/gnh124 PMC519125 15358833

[60] B. Chapman , J. H. Han , H. J. Lee , I. Ruud , and T. H. Kim , “Targeted Modulation of Chicken Genes In Vitro Using CRISPRa and CRISPRi Toolkit,” Genes 14 (2023): 906. 37107664 10.3390/genes14040906 PMC10137795

[61] J. K. Nuñez , J. Chen , G. C. Pommier , et al., “Genome‐wide Programmable Transcriptional Memory by CRISPR‐based Epigenome Editing,” Cell 184 (2021): 2503–2519.e17. 33838111 10.1016/j.cell.2021.03.025 PMC8376083

[62] Å. Segerstolpe , A. Palasantza , P. Eliasson , et al., “Single‐Cell Transcriptome Profiling of Human Pancreatic Islets in Health and Type 2 Diabetes,” Cell metabolism 24 (2016): 593–607. 27667667 10.1016/j.cmet.2016.08.020 PMC5069352

[63] H. E. Hohmeier , H. Mulder , G. Chen , R. Henkel‐Rieger , M. Prentki , and C. B. Newgard , “Isolation of INS‐1‐derived Cell Lines With Robust ATP‐sensitive K+ Channel‐dependent and ‐independent Glucose‐stimulated Insulin Secretion,” Diabetes 49 (2000): 424–430, 10.2337/diabetes.49.3.424. 10868964

[64] M. L. Maeder , S. L. Linder , V. M. Cascio , Y. Fu , Q. H. Ho , and J. K. Joung , “CRISPR RNA‐guided Activation of Endogenous Human Genes,” Nature Methods 10 (2013): 977–979. 23892898 10.1038/nmeth.2598 PMC3794058

[65] J. P. Concordet and M. Haeussler , “CRISPOR: Intuitive Guide Selection for CRISPR/Cas9 Genome Editing Experiments and Screens,” Nucleic Acids Research 46 (2018): W242–W245. 29762716 10.1093/nar/gky354 PMC6030908

