# Supplementary material for: Nanopore Electroporation: A New Delivery Method Within the Field of Epigenetic Editing
Source: Small. 2026 Mar 26;22(28):e13858. doi: 10.1002/smll.202513858 (PMC13181526; doi:10.1002/smll.202513858)
Supplement: Supplementary file 1 — Supporting File: smll73191‐sup‐0001‐SuppMat.docx. [file SMLL-22-e13858-s001.docx]

Nanopore Electroporation: A new delivery method within the field of epigenetic editing.

Supporting info

Frida Ekstrand^[[1]](#footnote-1)^#, Sabrina Ruhrmann^2^#, Karl Bacos^2^, Sabine Bartel^3^, Pytrick Jellema^3^, Marianne G. Rots^3^, Charlotte Ling^2^, Christelle N. Prinz*^1,4^.

1. Division of Solid State Physics and NanoLund, Lund university, Lund, Sweden

2. Epigenetics and Diabetes Unit, Department of Clinical Science in Malmö, Lund University, Scania University Hospital, Malmö, Sweden

3. Department of Pathology and Medical Biology, University of Groningen, University Medical Center Groningen, Groningen, the Netherlands

4. SciLifeLab, Lund University, Sweden

# Contributed equally to this work

* corresponding author: Christelle N. Prinz; Email: [Christelle.Prinz@ftf.lth.se](mailto:Christelle.Prinz@ftf.lth.se)


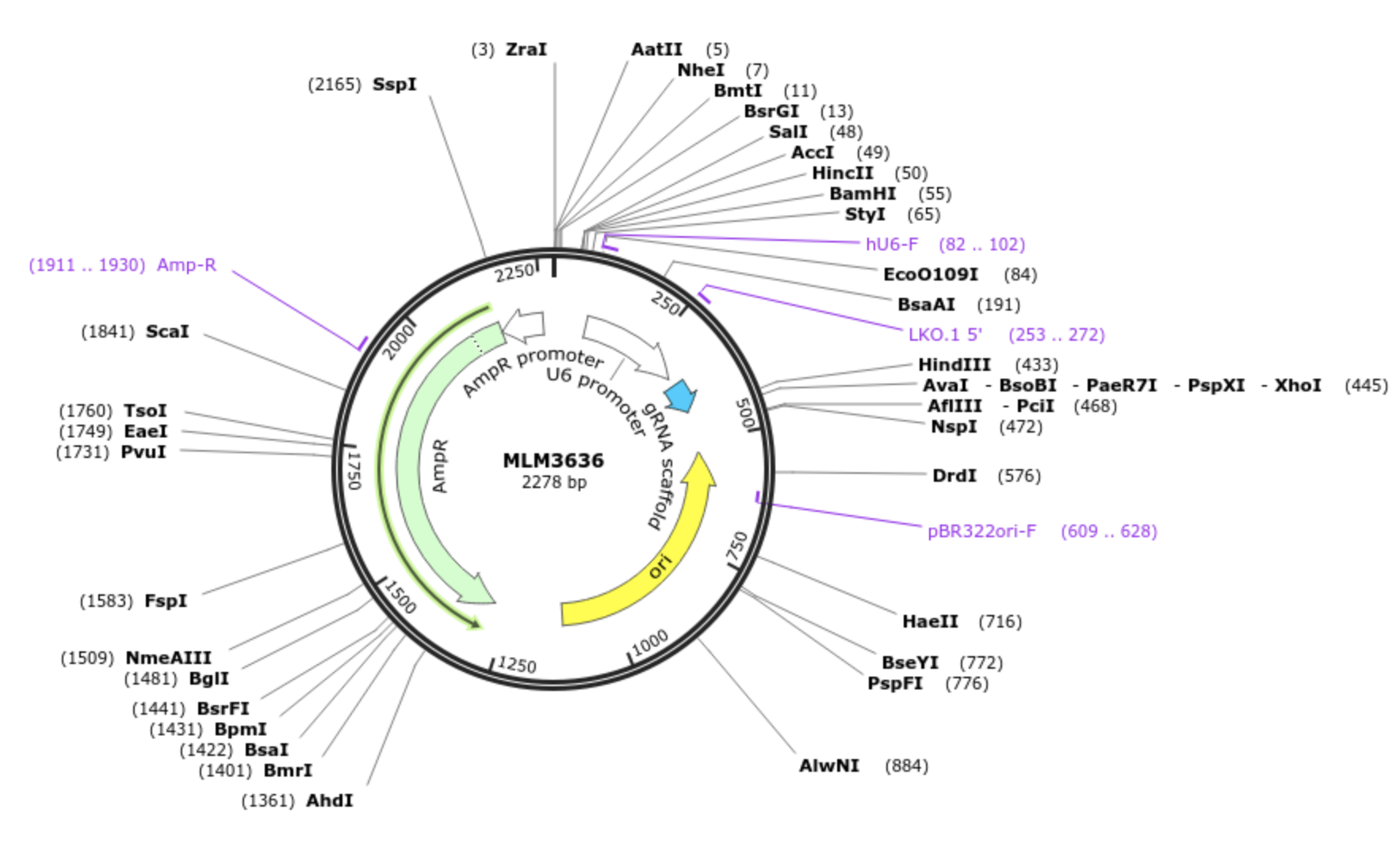


Fig. S1. Plasmid map of the sgRNA plasmid


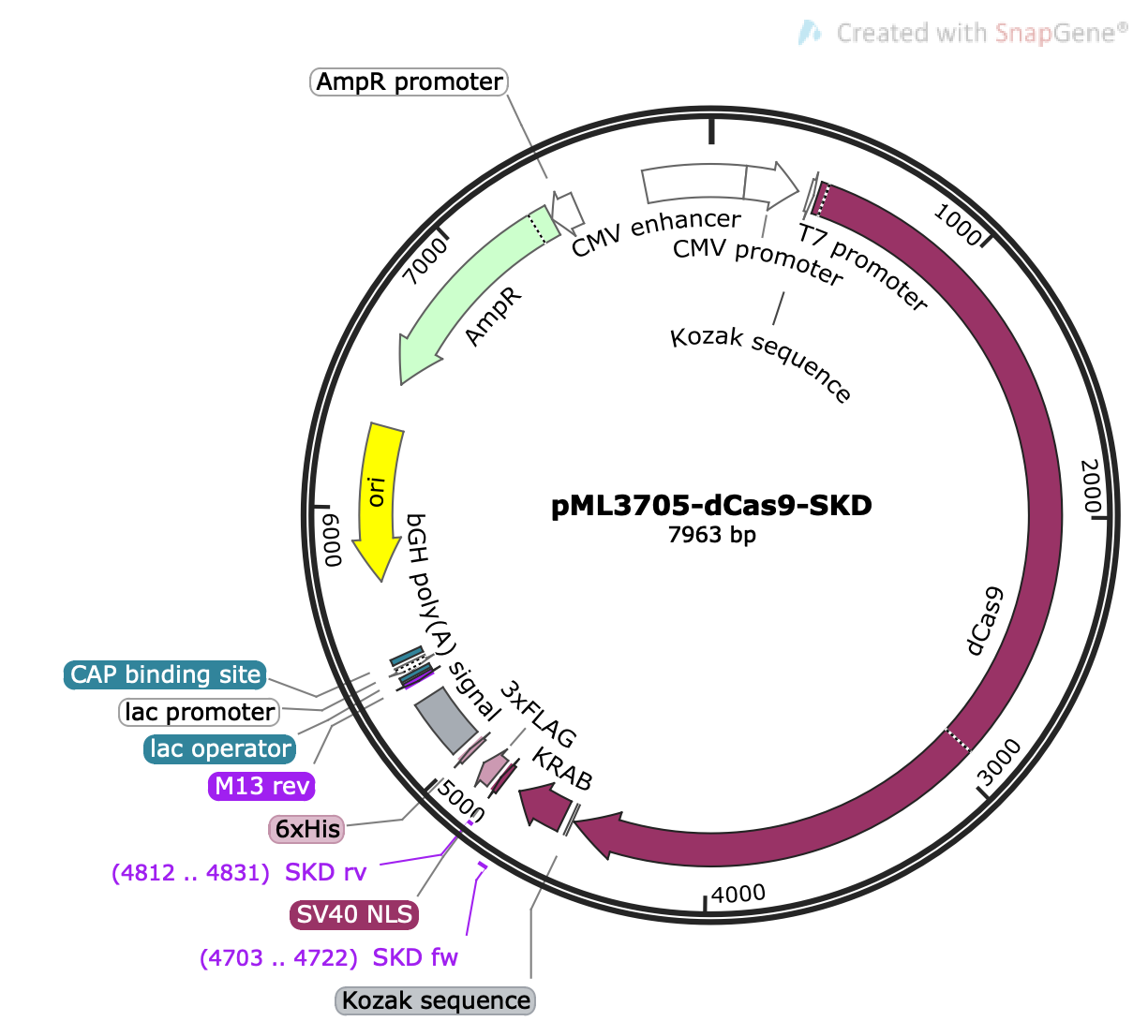


Fig. S2. Plasmid map of the dCas9-SKD plasmid.

**
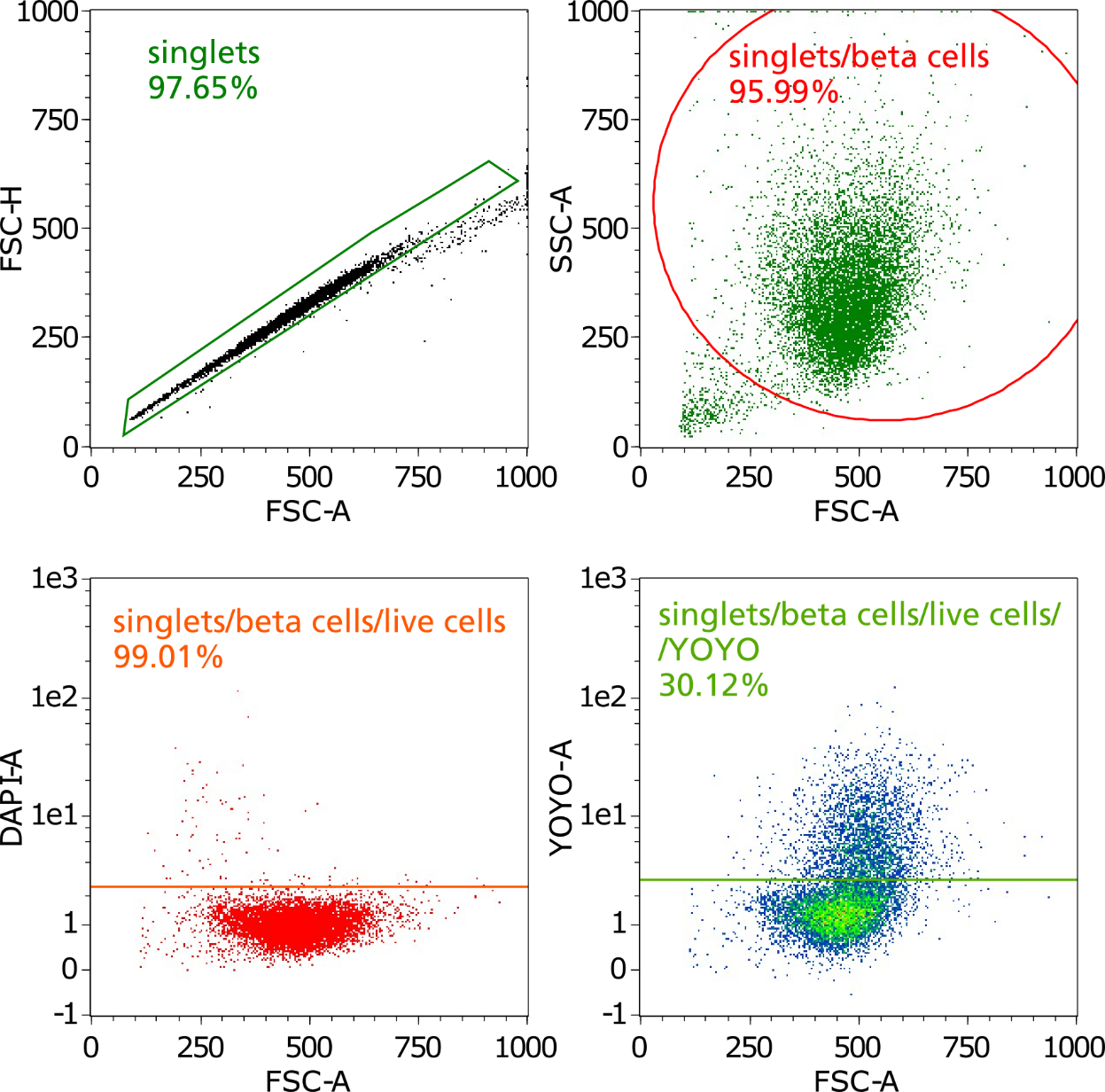
**

**Fig. S3**. *Gating strategy for assessing transfection efficiency after injection of plasmids stained with YOYO-1. Top left panel: selection of single cells; top right panel: Exclusion of debris; bottom right panel: Exclusion of dead cells; bottom righ panel: Percentage of transfected single live cells. FSC-A: Area of the forwards scatter signal; FSC-H: Height of the forwards scatter signal; SSC-A: Area of the side scatter signal; DAPI-A: Area of the dapi fluorescence signal; YOYO-A: Area of the YOYO-1 fluorescence signal.*

**
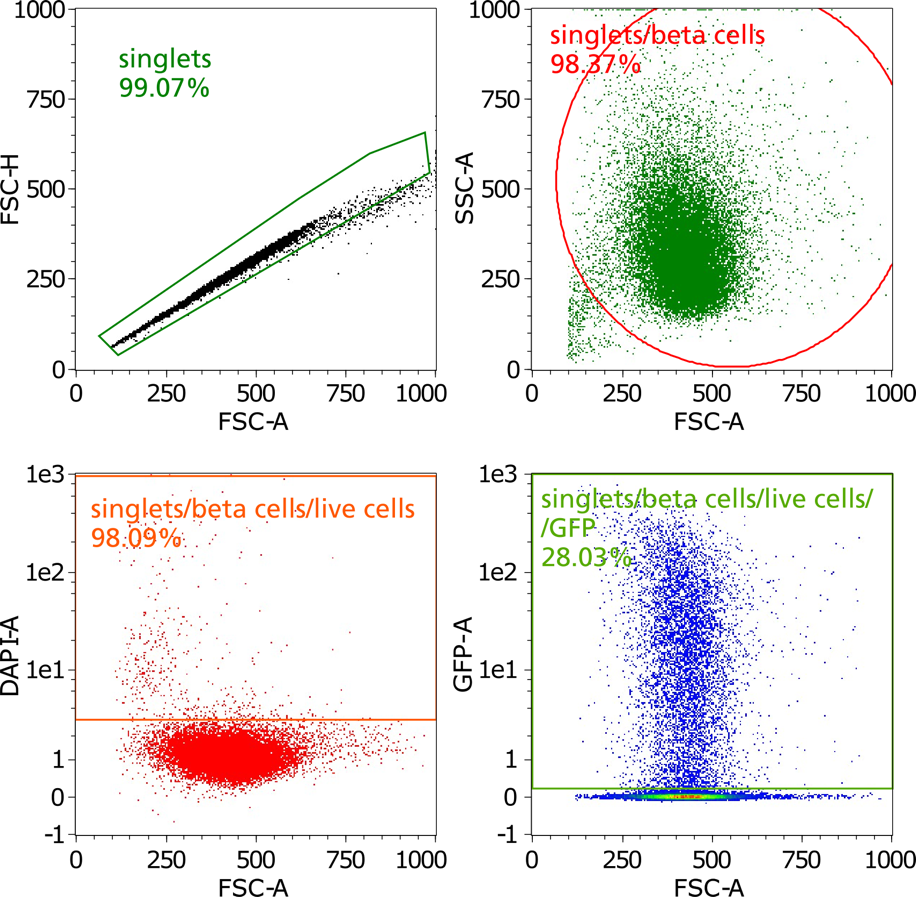
**

Fig. S4. *Gating strategy for assessing* *GFP expression after transfection of plasmid*. Top left panel: selection of single cells; top right panel: Exclusion of debris; bottom right panel: Exclusion of dead cells; bottom righ panel: Percentage of GFP-expressin single live cells. FSC-A: Area of the forwards scatter signal; FSC-H: Height of the forwards scatter signal; SSC-A: Area of the side scatter signal; DAPI-A: Area of the dapi fluorescence signal; GFP-A: Area of the GFP fluorescence signal.


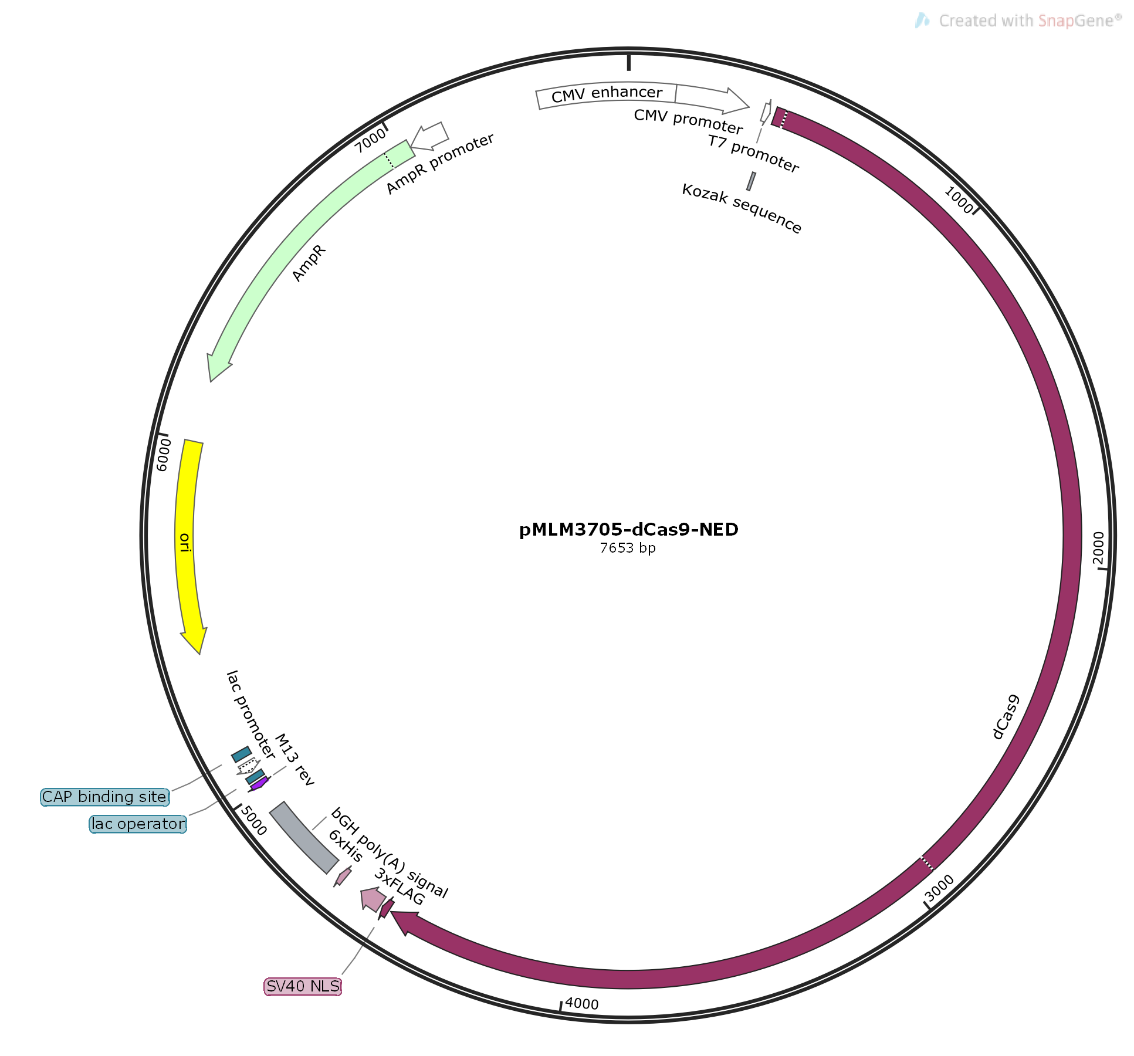


Figure S5. Plasmid map of the dCas9-NED plasmid.

*
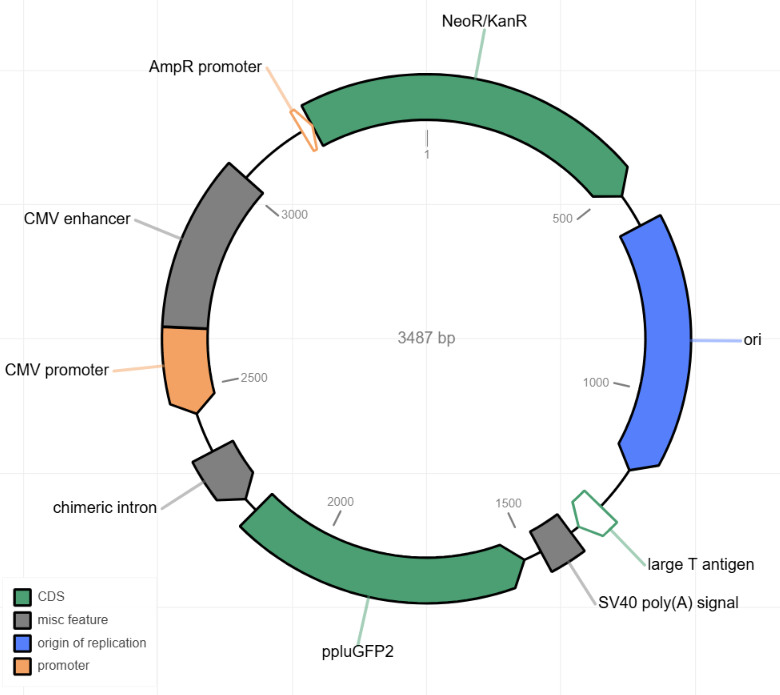
*

Figure S6. Plasmid map of the pMAX plasmid.

**Table S1.** *List of different transfection methods available for DNA plasmids, including viral transduction cationic lipids, and physical methods*

| **Transfection method** | **Cargos/Cell type** | **Advantages** | **Disadvantages** | **References** |
| --- | --- | --- | --- | --- |
| Cationic lipids  (Lipofection) | DNA plasmids, mRNA, siRNA  Broad range of cell lines and primary cells. | Can deliver various cargos to many cell types.  Inexpensive to produce. | Varying efficiency.  Endosomal entrapment.  Short duration of gene expression. | (1–3) |
| Lentivirus | DNA transgenes, RNA, plasmids (size limited).  Broad range of cell lines and primary cells and non-dividing cells such as neurons. | Enter nucleus in non-dividing cells.  Can transduce many cell types.  Stable transfection. | Risk of integration and mutagenesis. | (1–3) |
| Adenovirus | DNA transgenes, RNA, antigens.  Broad range of cell lines, primary cells including non-dividing cells. | Enter nucleus in non-dividing cells. | Risk for evoking immune response and mutagenesis.  Transient transfection only. | (4–6) |
| Adeno-associated virus | Therapeutic genes, RNA molecules.  Broad range of primary cells and tissue, including non-dividing cells. | Enter nucleus in non-dividing cells.  Not incorporating itself into the target cell genome.  Evoke milder immune response than other viruses.  Nonpathogenic. | Smaller packaging size.  Transient transfection only. | (4–6) |
| Bulk Electroporation | Plasmids, RNA, nanoparticles, proteins.  Broad range of cell lines and primary cells. | High throughput.  Work on many cell types. | Low viability.  Varying efficiency.  Electric field affects the whole cell membrane. | (3, 7, 8) |
| Microinjection | Plasmids, RNA, nanoparticles, proteins.  Broad range of cell lines and primary cells, including neurons and embryos. | Efficient.  Can deliver directly into the nucleus – fast gene expression  Controllability. | Low throughput.  Require experience to perform. | (3, 9) |

**References**

1. Z. X. Chong, S. K. Yeap, W. Y. Ho, Transfection types, methods and strategies: A technical review. *PeerJ* 9 (2021).

2. S. Tong, B. Moyo, C. M. Lee, K. Leong, G. Bao, Engineered materials for in vivo delivery of genome-editing machinery. *Nat Rev Mater* 4 (2019).

3. A. K. Fajrial, Q. Q. He, N. I. Wirusanti, J. E. Slansky, X. Ding, A review of emerging physical transfection methods for CRISPR/Cas9-mediated gene editing. *Theranostics* 10 (2020).

4. A. Fus-Kujawa, *et al.*, An Overview of Methods and Tools for Transfection of Eukaryotic Cells in vitro. *Front Bioeng Biotechnol* 9 (2021).

5. M. S. Al-Dosari, X. Gao, Nonviral gene delivery: Principle, limitations, and recent Progress. *AAPS Journal* 11 (2009).

6. D. Pei, M. Buyanova, Overcoming Endosomal Entrapment in Drug Delivery. *Bioconjug Chem* 30, 273–2 (2019).

7. T. Kotnik, *et al.*, Electroporation-based applications in biotechnology. *Trends Biotechnol* 33 (2015).

8. G. W. Lee, *et al.*, Nanoporous electroporation needle for localized intracellular delivery in deep tissues. *Bioeng Transl Med* 8 (2023).

9. Y. T. Chow, *et al.*, Single cell transfection through precise microinjection with quantitatively controlled injection volumes. *Sci Rep* 6 (2016).

1. [↑](#footnote-ref-1)
